# The role of grandparents in childhood obesity in China - evidence from a mixed methods study

**DOI:** 10.1186/s12966-015-0251-z

**Published:** 2015-06-30

**Authors:** Bai Li, Peymané Adab, Kar Keung Cheng

**Affiliations:** College of Medical and Dental Sciences, University of Birmingham, Edgbaston, Birmingham, B15 2TT UK

**Keywords:** Child care, Childhood obesity, Chinese children, Eating behavior, Health behavior, Body mass index

## Abstract

**Background:**

The current literature on the influences of family environment on childhood obesity is predominantly based on western populations and has focused on the role of parents. This study examined the influence of grandparents on the development of obesity among Chinese primary school aged children.

**Methods:**

A mixed methods study was conducted in four socioeconomically distinct primary school communities in two cities of southern China. The qualitative study (17 focus groups and four personal interviews) involved parents, grandparents, school staff, and food retailers in the vicinity of the schools (*n* = 99) and explored perceived causes of childhood obesity. The cross-sectional study examined the association between children’s objectively measured weight status and reported health behaviours, and the presence and role of grandparents in the household. It included children from three randomly selected third grade (8 to 10 years) classes from each school (*n* = 497).

**Results:**

Grandparents were commonly perceived to contribute to childhood obesity through inappropriate perception (e.g. fat children are healthy and well cared for), knowledge (e.g. obesity related diseases can only happen in adults; the higher the dietary energy/fat content, the more nutritious the food), and behaviour (e.g. overfeeding and indulging through excusing the children from household chores). Conflicting child care beliefs and practices between grandparents and parents, and between grandparents and school teachers, were felt to undermine efforts to promote healthy behaviours in children. In the cross-sectional study, children who were mainly cared for by their grandparents were more likely to be overweight/obese (adjusted OR = 2.03; 95 % CI = 1.19 to 3.47); and to consume more sugar-added drinks and unhealthy snacks (*B* = 2.13, 95 % CI = 0.87 to 3.40), than children who were mainly cared for by their parents or other adult. Children who lived with two or more grandparents in the household were more likely to be overweight/obese than children who did not live with any grandparent (adjusted OR = 1.72; 95 % CI = 1.00 to 2.94).

**Conclusions:**

Involvement of grandparents in childcare is an important factor contributing to childhood obesity in China. Future preventive interventions should include strategies that target grandparents.

## Background

Childhood obesity is a pandemic, associated with physical and psychological morbidity in children, as well as contributing to an increase in non-communicable chronic diseases (NCDs) and premature mortality in adulthood [1]. Over the last 2 decades, the rate of increase in childhood obesity prevalence in mainland China, particularly in urban centres, exceeds the trends seen in many other countries [2]. A dietary shift from high-carbohydrate to high-fat, high-energy dense foods, and related increase in rates of non-communicable diseases has been well documented in China [3–5]. One important contributor is the lag in health-related (particularly nutrition-related) knowledge and perceptions among the general population, compared with the speed of change in social and economic conditions [6–11]. This is particularly common among the older generation who experienced underweight, under-nutrition, food shortage, physical hardship and deprivation in their early lives before China’s economic reform. This older generation are the grandparents of the current cohort of Chinese children, predominantly in single child families (widely known as the single family treasure). China introduced its One-Child Family Planning Policy in late 1979 and there are nearly 150 million single-child households in the country [12, 13]. Culturally, these grandparents are held in great respect and often live in three-generation households. More importantly, they are usually the main child carers in cross-generation households.

The influence of the family and home environment on children’s health behaviours is well established. Parenting style, such as rewarding desirable child behaviours using unhealthy foods [14–16] or with sedentary screen based activities [17], the influence of parental behaviour modelling [18–27], the number of screen media present in the home [24] and family meal habits [28–30] have all been shown to be associated with children’s dietary and physical activity behaviours. A few studies have also reported an association between family structures and children’s health behaviour. For example, cross-sectional analysis of data from 3798 seventh grade American adolescents showed that self reported physical activity level were higher in boys who lived with a mother, compared with those who did not [17]. A 3-year prospective study in 279 Swedish adolescents (aged 12 years at baseline) found that girls living in households where the parents were divorced, had more weight control related concerns, compared to those living with both parents [31]. Furthermore, the most recent systematic review of childhood obesity prevention trials [32] suggested that interventions focusing on modifying the home environment are worth pursuing. However, the current literature is predominantly based on western populations and the focus of the family environment has been the role of parents in obesity prevention. In many countries, particularly in China, grandparents are key providers of child care. Yet their contribution to the childhood obesity epidemic, and their role in prevention strategies, has rarely been considered.

We conducted a mixed methods study to investigate the impact of grandparents on the childhood obesity epidemic in China, in order to inform the development of culturally relevant childhood obesity intervention programmes.

## Methods

This mixed methods study includes both a qualitative and quantitative element. While the former gives greater depth, the latter offers greater breadth, so inferences from both parts of the study can be triangulated (to confirm) and complement each other [33]. First, we explored the perceived causes of childhood obesity among a range of stakeholders using focus groups and in-depth interviews (described in the Qualitative methods below). We focused the analysis on themes related to the role of grandparents in Chinese households, and their influence on children’s health behaviours. We then used a cross-sectional study (described in the Cross-sectional method below) to examine the relationship between the role of grandparents in child care and children’s weight status, diet and physical activity habits. The findings from both the qualitative and quantitative elements are drawn together in the discussion.

Ethical approval for both parts of the study was obtained from the Life and Health Science Ethical Review Committee at the University of Birmingham.

### Qualitative methods

#### Setting

The qualitative study was conducted between December 2009 and May 2010 in two cities of southern China: Guangzhou (GZ) and Hechi [34]. Data collection took place in four socioeconomically distinct primary school communities. Three in GZ, comprising low, middle and affluent class communities and one affluent class community in HC. The local Health Bureaus use these as sample schools for routine student health monitoring, to represent the diversity of pupils, and the socioeconomic status of the school communities are defined by them using measures such as annual average household expenditure and income. We conducted focus groups and interviews with a range of participants relevant to children, in each of the school communities to explore perceived contributors to childhood obesity.

#### Participant selection and recruitment

We identified key stakeholders relevant to children for each school community. Participants were invited by identity group to attend focus groups, to ensure discussion of shared experiences [35]. These included separate groups for i) parents; ii) grandparents; iii) class-level teachers and school nurses; iv) PE teachers; v) catering staff responsible for children’s school meals and shop retailers. Inclusion of head teachers in the same focus group as other school staff was likely to introduce a power imbalance and may have impeded discussion. They were therefore invited for individual interviews, following a similar topic guide to that used in the focus groups. All identity groups were included for each of the three school communities in GZ, but only family member focus groups and interview with the head teacher were conducted in HC. This was mainly a pragmatic decision based on available resources, but also because the focus of data collection in HC was to explore whether new contradictory or complementary information would be obtained from families in a different urban centre. Given the highly centralised and regulated education system in China, variation in experiences of school staff in different centres was considered to be low.

School teachers assisted in purposively identifying and recruiting participants in their respective school communities following a pre-defined recruitment strategy that aimed to maximise gender balance and the diversity of participants’ socioeconomic backgrounds. Informed consent (with information on study purpose and the roles of voluntary participants) was sought for all participants. A minimum of five participants with at least two males and two females took part in each of the 17 focus groups.

#### Focus group and interview process

**Fig. 1:**
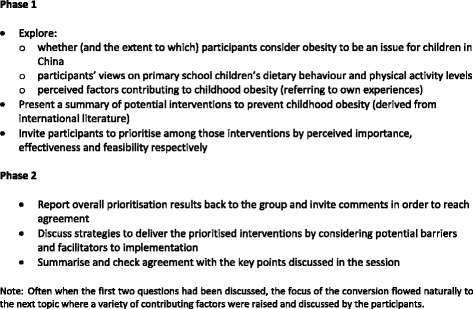
Summary of focus group topic guide

All focus groups (*n* = 95 participants) were held within the schools and consisted of two sessions, each lasting for approximately 90 min (Fig. 1 summarises key discussion topics and activities). They were moderated by an experienced native Chinese speaker (BL) and all sessions were audio recorded. The main purpose of the first session was to explore perceived contributory factors of childhood obesity in China. The secondary objective was to facilitate participants through a process of prioritising potential components for a childhood obesity prevention intervention. The second session aimed to explore preferred strategies for the delivery of interventions. In this paper we focus on findings related to perceived causes of childhood obesity.

One to one in-depth interviews (*n* = 4) with school principals were run at the offices of the principals and each lasted for around 45 min. Discussions focused on the broad topic of perceived causes of child obesity. To better enable discussions from the unique perspective of school leaders, questions were phrased as ‘barriers’ that schools had encountered in promoting healthy eating and physical activity on campus and ‘what schools want/need’ for playing a better role in children’s healthy behaviour promotion (Table 1 summarises the topic guide for the interviews).

**Table 1:** Summary of interview topic guide

	Objectives	Questions
Introduction (5 min)	Warming up questions, to explore school Principal’s understanding of childhood obesity	1. What do the terms ‘overweight children’ and ‘obese children’ mean to you?
		2. Do you think that overweight/obesity is an issue for children in China?
		3. What about in your community, is it an issue here?
Topic 1 (5–10 min)	To gain insights into the Principal’s perceptions of the causes of childhood obesity	1. What do you think of primary school children’s dietary behaviour/pattern/habits in general?
		2. What do you think of primary school children’s physical activity levels in general?
Topic 2 (5–10 min)	To understand the extent to which the Principal feels that schools should play an active role in promoting and supporting healthy eating and physical activity for the aim of preventing/controlling childhood obesity/overweight?	1. To what extent, do you think primary schools should play an active role in promoting healthy eating and physical activity in children?
		2. Why?
Topic 3 (15 min)	To explore school’s current activities regarding healthy eating and/or physical activity promotion and the barriers encountered in implementing the activities	1. Does your school currently have any programme? If any, probe what/how they are going. If none, go directly to Topic 4.
		2. Has the school met any difficulties or barriers in implementing the programme(s)?
Topic 4 (15 min)	1. To understand what schools want/need for playing a better role in children’s healthy behaviour promotion	1. What kind of resources or support, do you think would make it easier for your school to take an active role?
		Probe: Have you had any experience in using these resources or support? How was the experience?
	2. Summarising key points discussed and arisen to ensure data credibility	

#### Coding and analysis

All audio records from the focus groups and interviews were transcribed verbatim in Mandarin. Thematic analysis of data gathered from the first phase was conducted to develop an in-depth understanding of factors perceived to be contributing to childhood obesity in China. Transcripts were systematically and thoroughly read by BL and initially coded in Mandarin using NVivo 8 computer package. Illustrative quotes were translated by BL who moderated the sessions to ensure the accuracy of contextual and cultural meanings. In discussion with PA, emerging themes were identified using an inductive process, and a preliminary coding framework was developed in English using transcripts from five focus groups and one interview. Constant comparison was used to further develop and refine the initial coding framework, as the remaining data were coded [36, 37]. This was an iterative process involving continuous discussions between BL and PA. By examining the differences and relationships among created themes and codes within and across transcripts of various stakeholder groups, we established the final analytical categories. These are reported in the Result sections.

### Cross-sectional methods

#### Setting

In the same academic year, we conducted the cross-sectional study in the same communities as the qualitative study to enable data triangulation and complementarity.

#### Study sample

With permission from local Education Bureaus and informed consent from children’s parents we obtained weight and height data for third-year students (aged 8–10) who were in the same schools as those from which we recruited the participants for our qualitative study. This data was collected as part of the routine student health surveillance programme, in the same school year. Measurements were undertaken by trained health professionals working for the Education Bureaus, following standardised anthropometry measurement protocols [38]. In each school, three (out of up to 6) classes of third year students were randomly selected (using a random number generator) to be included in the study. In addition, the parents of all selected children were invited to complete a questionnaire which collected information on family, neighbourhood, socio-demographic and obesogenic- behavioural characteristics of Chinese primary school children. The details of the questionnaire and its design were described previously [39]. In brief, the questionnaire included questions regarding the household composition, the children’s diet (using adapted Health Behaviour in School-Aged Children food-frequency questionnaire [40]) and physical activity levels (using the Godin Leisure Time Questionnaire [41]).

#### Assessment of the role of grandparents in childcare

We asked two questions specifically about the role of grandparents. First, we asked whether any grandparents live in the household, and secondly, whether grandparents play a role in caring for children (by asking who the child spends most of their time with after school during the weekdays), irrespective of whether or not they live in the household.

#### Assessment of outcomes and statistical analysis

The main outcome was child weight status. Children were categorised into overweight/obese and non-overweight groups using the age and sex specific WHO 2007 child growth reference values [42]. The dietary habit questions were used to estimate the frequency of consumption of unhealthy snacks as the sum of parent reported average servings of salty high fat snacks, candies and sugared beverages over the previous week. The average amount of time spent by children in moderate to vigorous physical activity (MVPA) each day was estimated by averaging the reported typical week and weekend day activities. Children were categorised into whether or not they engaged in recommended levels of MVPA (at least 60 min per day).

Multivariable regression analysis was used to examine the relationships between the presence and role of grandparents and 1) child weight status, 2) frequency of unhealthy snack consumption and 3) the likelihood of engaging in a daily minimum of 60 min MVPA. As well as socio-demographic variables, including child sex, age in months, and mother’s education level (proxy measure of socioeconomic position), we adjusted for school (as a fixed effect) and birth weight (shown prospectively to be related to child weight status) [43, 44] in all analyses. We carried out two sensitivity analyses; first by using the Chinese national reference norm [45] to define overweight and obesity instead of the WHO 2007, and secondly by repeating the analyses, excluding the children in the underweight category.

## Results

### Qualitative results

#### Participant characteristics

Ninety five participants (42 male) took part in 17 focus groups (15 in GZ) and four school principals participated in the personal interviews (Table 2). No new information emerged after the 15th focus group and the 3rd interview.

**Table 2:** Summary of the characteristics of study participants

Site	Parents	Grandparents	Teacher & school nurses	PE teachers	Retailer/catering staff	School principal
GZ	18(9M;9F)	18(9M;9F)	15(1M;14F)	16(9M;7F)	15(6M;9F)	3(F)
HC	7(5M;2F)	6(3M;3F)	0	0	0	1(F)
Total	25(14M;11F)	24(12M;12F)	15(1M;14F)	16(9M;7F)	15(6M;9F)	4(F)

Figures represent numbers of participants in different types of focus groups and cities

*M* male, *F* female

Around half of the parents who participated in the focus groups indicated that they had at least one grandparent living in their households and playing an important role in child care. The role and influence of grandparents on children’s health behaviour was discussed by almost all groups, based on either direct experience, or knowledge of others. Relevant themes and some illustrative quotes are presented below.

#### Preference for fat children

A recurrent theme in all groups was an affection and preference for fat children among grandparents.

*‘In the past, they had underweight, under nutrition and food was not enough…families were also poorer…now fat children are viewed as healthy, strong and well cared for’* (Parents from affluent class in GZ)*.*

*‘Fat means wealthy’* (Grandparents from HC)*.*

This belief among grandparents often conflicted with the views of parents who did not always share the sentiments:

*‘My mother loves seeing my daughter getting big…I said not so much meat and snacks and she should learn swimming but she doesn’t understand and thinks I am wrong…’* (A mother from middle class in GZ)*.*

*‘I said to my son you need to lose weight, he replied that grandma said I am just strong…only you want me to’* (A mother from lower class in GZ).

#### Poor recognition of childhood obesity

Another common theme in all groups was the lack of recognition of obesity and overweight in children, particularly among grandparents. Assessment of weight status was often done by comparison with other children, and norm setting was among their peers. Many participants considered the annual measurement of children in schools to be meaningless, and they did not understand the reports sent home. As one father commented:

*‘We don’t understand what the figures mean …we can’t influence grandparents’ opinion when they believe that the overweight kid is normal weight; but a formal health report may do’.*

#### Poor knowledge of health consequences of childhood obesity

Another common theme emerging from the parent and teacher FGs was the belief that grandparents know little about the health consequences of overeating. Similarly they are unaware of the benefits of a healthy diet. One mother from a middle class area in GZ city said:

*‘My parents feed lots of meat to my daughter who is already overweight. They say the “3 highs”* (high blood sugar, high blood pressure and high cholesterol) *do not affect children, as they are the burden of adults…children can eat freely’.*

#### Misperceptions about healthy diet

Parents and teachers (with children) from all sample schools shared the belief that grandparents hold a mistaken perception of what constitutes a healthy diet in children.

*‘I told my boy his diet needs some improvement…my mum said she is happy with his diet, pretty healthy…not picky, not wasteful… eats almost everything…eats enough meat and enough oil is used in cooking…’. ‘In their time, meat and oil were treasures so now they feel the more the better’. ‘I decided to move out with my wife and son…his grandparents were a big problem…we couldn’t change anything when we lived together’.*

#### Family structure and influence of grandparents

The single-child family structure was discussed by all as a contributor to obesogenic behaviours. Family members and school staff commented that having only one child at home makes the promotion of healthy eating difficult especially when a grandparent is living with them. As one parent said:

*‘In my childhood, sisters and brothers fought to eat at meal times simply because there were so many children but insufficient food…now each child is cared for by 4 to 6 adults… the single family treasure receiving too much love and attention…I wish to change this situation but it is hard to avoid overfeeding the child by grandparents’.*

It was frequently discussed that grandparents overindulge their single grandchildren, thus undermining the efforts of parents and school teachers in promoting healthy eating. One parent noted:

*‘They buy snacks for the child when he/she has behaved well…sometimes in a secret way as I don’t allow snacking…but I can’t manage my parents’.*

And a teacher commented:

*‘When collecting children, grandparents often bring a sweetened drink or dessert…we educate our pupils don’t snack too much…children remember they will get something after school so some of them don’t eat school lunch properly’.*

In addition to the influence on dietary habits, grandparents were considered to over protect grandchildren from physical household chores, thus limiting their activity levels. Several parents made comments on this issue:

*‘Now a 9 year old child doesn’t know how to make his own bed…he knows grandma wouldn’t let him do this’. ‘My parents told me how many physical tasks they undertook in childhood; so let the only child enjoy a pain-free childhood…I should respect parents’ opinion’.*

### Cross-sectional results

#### Sample characteristics

Questionnaires were completed by 508 of 554 parents invited (response rate = 91.7 %). A total of 497 child/parent dyads were included in the analyses. Among all the participating children, 228 (45.9 %) of them lived with at least one grandparent, and 87 (17.6 %) were mainly cared for by a grandparent (i.e., a grandparent spent the longest time with the child outside of school time during weekdays). These patterns varied by child socioeconomic status and sex. Live-in grandparents were more common among families with more educated mothers compared to those where mothers had no higher degree (48.5 versus 42.0 %) and among boys compared with girls (49.4 versus 42.1 %). Grandparents as carers were more common among girls compared with boys (18.4 versus 16.9 %) and among families with less educated mothers compared to those where mothers had higher degree (20.8 versus 15.3 %). Overall, approximately a quarter of the children were overweight or obese. Among the four socioeconomically distinct sample schools, the combined prevalence was higher in the upper socioeconomic communities than in the middle and lower socioeconomic ones.

#### Grandparents and child weight status and health behaviours

Living with at least two grandparents in the same household (adjusted OR = 1.72, 95 % CI: 1.00–2.94) and being primarily cared for by grandparent(s) (adjusted OR = 2.03, 95 % CI: 1.19–3.47) were significantly associated with child overweight/obesity (Table 3). There was also a statistically significant trend for higher frequency of unhealthy snack consumption among children who had grandparent(s) as the main carer (non-standardised coefficient *B* = 2.13 and standardised coefficient Beta = 0.15). Thus, children who were mainly cared for by a grandparent consumed over two more servings of unhealthy snacks per week in comparison with children who were mainly cared for by their parents or other adults. In terms of physical activity, we did not find any association between the role of grandparents and children’s likelihood of engaging in a least 60 min of daily MVPA.

**Table 3:** Relationship between presence of grandparents in the household, and child weight status and health behaviours

Outcome variable: risk for being overweight or obese		
Explanatory variables	Unadjusted OR (95 % CI)	Adjusted OR (95 % CI)
Presence of grandparents in the household		
0	1.00	1.00
1	1.08(0.65–1.78)	1.09(0.65–1.83)
2/more	1.70(1.02–2.83)*	1.72(1.00–2.94)*
Main child carer		
Mother/father/other	1.00	1.00
Grandmother/grandfather	1.76(1.06–2.91)*	2.03 (1.19–3.47)**
Outcome variable: weekly consumption frequency of unhealthy snack		
Explanatory variable	Coefficient Beta	Coefficient B (95 % CI)
Main child carer		
(A grandparent, compared with a parent or other adult)	0.15	2.13(0.87–3.40)**
Outcome variable: child’s likelihood of engaging in at least 60 min MVPA a day		
Explanatory variable	Unadjusted OR (95 % CI)	Adjusted OR (95 % CI)
Main child carer		
Mother/father/other	1.00	1.00
Grandmother/grandfather	0.78(0.40–1.52)	0.75(0.38–1.50)

MVPA denotes moderate to vigorous physical activity. Child sex, birth weight, child age in months, school and mother’s education level were adjusted for in the first two models (for the first outcome measure). The same co varieties (excluding birth weight) were controlled for in the last two models (for the remaining two outcome measures)

*denotes *P* ≤ 0.05; **denotes *P* ≤ 0.01

#### Sensitivity analyses

We repeated the analyses for the two exposures associated with child weight status using the WGOC instead of the WHO 2007 reference standard and found no material difference to the findings. In addition, we repeated the analyses in non-underweight children only. This also made no difference to the main findings.

## Discussion

### Summary of principal findings

In this mixed methods study we found the influence of grandparents on childhood obesity to be a dominant theme. In the qualitative study, all groups considered grandparents to play a significant role in the development of childhood obesity, through unhealthy attitudes (e.g. belief that fat children are healthy and well cared for), indulging behaviour (e.g. overfeeding and sparing the ‘single family treasure’ from household chores) and poor health knowledge (e.g. not understanding the health consequences of obesity in children). They also counter the attempts by parents and school staff to promote healthy eating and physical activity in children. These attitudes and beliefs largely stem from the historical context of famine and poverty in China during their childhood, where being slim represented poverty and poor health. In support of the qualitative findings, having grandparents living in the household and grandparents taking on a main carer role for children were both associated with increased risk of overweight/obesity in our cross sectional sample. Children who were mainly cared for by a grandparent also consumed unhealthy snacks and drinks more frequently.

### Findings in relation to previous literature

Our findings show that grandparents are an important influence on children’s weight status, partly through limited understanding of the consequences of and risk factors for obesity and also through a desire to over-indulge their single grandchildren. This is in keeping with findings from a previous small qualitative study in Beijing, which reported that within three-generation households, grandparents overfed and pampered grandchildren through food [46]. The study focused on pre-school children, and did not consider the wider influence of grandparents on children’s health behaviours. Our findings are also in keeping with those reported from a cohort study in Hong Kong which found that informal child care (including care by paid domestic helpers and other family members such as grandparents), when compared with parents as main carers, was associated with overweight/obesity in children [47]. Similar findings have also been reported in the UK [48], Canada [49] and the US [50], where pre-schoolers cared for by a relative were found to be at increased risk of overweight. The influence of grandparents was also implicated as an important contributor to childhood obesity in South Asian families who often live in multi-generation households [51].

The phenomenon that Chinese children are exempted from household chores but under high expectation to perform well academically was also reported in a previous cross-sectional study [52]. This can be partly explained by the long-standing examination-oriented education system in China that has resulted in physical activity opportunities being squeezed out at school and within the home [53, 54]. Grandparents who are seeing their ‘only family treasure’ living under considerable study pressure would therefore tend to protect their grandchildren from taking parts in household chores.

Most of the explanations for the mechanisms by which grandparents contribute to the childhood obesity epidemic are also similar to those reported in other studies. Poor knowledge of the health effects of overeating or unhealthy diet in children among carers has been implicated in qualitative studies among mothers in Australia and North America [55, 56]. Our finding of cultural preference for “fat” children among grandparents has also been reported elsewhere [51] and several qualitative studies across the world have identified poor recognition [55, 57, 58] and misinterpretation of children’s weight status [59] by carers as a problem.

### Strengths and limitations

This is the first qualitative study in China to explore the views of a wide range of stakeholders on causes of childhood obesity. Participants from a variety of socio-economic backgrounds provided views from a broad perspective and this allowed data saturation to be reached. Several steps were taken to ensure data trustworthiness, and we have provided a transparent description of the methods and analysis approach. The cross-sectional study used objective anthropometric measures to assess children’s weight status. A published estimation (based on WGOC reference norm) of overweight and obesity prevalence among Guangzhou’s 6- to 15-year students in the same year [60] was similar to our estimation within the study sample using the same definition, suggesting the sample was fairly representative. A major strength is the corroborative evidence from two different sources, suggesting that grandparents have an important influence on childhood obesity in urban China. The cross-sectional study validated and quantified the qualitative findings while the qualitative findings offered insights into and possible explanation for the mechanism behind the relationships identified in the cross-sectional study.

There were also limitations to this study. China is a vast country with varying geographic conditions and dietary traditions, thus our findings may not be generalisable to other parts of the country. One limitation of the cross-sectional study was that children’s diet and physical activity behaviours were assessed by parental reports. However, a review of methods for assessing physical activity patterns in children concluded that parent-proxy reports were more accurate than children’s self reports [61].

### Implications for future research and policy

Informal childcare, mainly provided through grandparents, is a growing social trend internationally [62]. In China, around half of urban families have grandparents involved in childcare. Our findings suggest that grandparents tend to indulge, overfeed and protect grandchildren in their care from physical chores, thus increasing their risk of obesity. The single child policy and historical deprivation experienced by the older generation were identified as important contributors. The underlying motive for the action of grandparents is affection for their treasured grandchild and stem from their personal experiences, misunderstanding and poor recognition of the adverse health effects of childhood obesity. Thus future studies should focus on how to channel the positive motives towards encouraging grandparents to promote healthy behaviours among children. Studies in other populations may also identify other contextual factors that may be influencing grandparent behaviours in other settings.

## Conclusions

This mixed-methods study has identified grandparents as important targets for obesity prevention interventions. Findings from the qualitative study indicate strategies and components that can be pursued within intervention programmes.
